# Reducing health inequalities through general practice in the UK: a realist review (EQUALISE)

**DOI:** 10.1093/eurpub/ckac129.030

**Published:** 2022-10-25

**Authors:** A Gkiouleka, G Wong, I Kuhn, S Sowden, F Head, C Bambra, R Harmston, S Manji, A Moseley, J Ford

**Affiliations:** 1 Public Health and Primary Care, University of Cambridge, Cambridge, UK; 2 University of Oxford, Oxford, UK; 3 Newcastle University, Newcastle Upon Tyne, UK; 4 NHS Cambridgeshire and Peterborough CCG, Cambridge, UK; 5 Patient and Public Involvement Representative, Cambridge, UK

## Abstract

**Background:**

In the UK, chronic conditions such as cancer, heart disease, stroke, and chronic obstructive pulmonary disease are driving health inequalities in life expectancy and were responsible for two-thirds of premature mortality in 2017. Voices that stress the importance of primary care in reducing health inequalities have been strengthening during the last decade. However, defining the most effective strategies to reduce health inequalities through general practice remains a challenge.

**Aims:**

This study examines the evidence on interventions in primary care that are likely to decrease inequalities in NCDs and especially cancer, diabetes, cardiovascular and chronic obstructive pulmonary disease and will provide healthcare organisations with guiding principles on what should be commissioned.

**Methods:**

The study is a realist review following Pawson's model. Based on a programme theory, we screened systematic reviews of interventions delivered in primary care and through their references, we identified primary studies reporting on inequalities across PROGRESS-Plus criteria. The data were analysed in light of the initial program theory and organised in a model informed by Collins’ Domains of Power framework.

**Results:**

Out of 251 included reviews we retrieved 6,555 primary studies which resulted in 333 studies for data extraction. We found that there are five guiding principles operating simultaneously across four different domains which can reduce health inequalities in General Practice. The principles include flexibility, continuity, inclusivity, intersectionality, and community and operate simultaneously across the domains of structures and policies; narratives and ideas; rules and practices; and relationships and experience.

**Conclusions:**

Flexibility, continuity, inclusivity, intersectionality, and community are the five principles which should guide the design and delivery of General Practice for the reduction of health inequalities.

**Key messages:**

• Flexibility, continuity, inclusivity, intersectionality, and community are the five principles which should guide the design and delivery of General Practice for the reduction of health inequalities.

• Action to reduce health inequalities should be taken simultaneously across the domains of structures and policies; narratives and ideas; rules and practices; and relationships and experience.

